# Effect of a Wireless Vital Sign Monitoring System on the Rapid Response System in the General Ward

**DOI:** 10.1007/s10916-022-01846-8

**Published:** 2022-08-26

**Authors:** Won Ho Han, Dae Kyung Sohn, Yul Hwangbo, Hee Jung Park, Mijung Kim, Yoona Choi, Il Won Shin, Jung Min Lee, Heungki Jeon, Ki Chung Ryu, Taesik Yoon, Jee Hee Kim

**Affiliations:** 1 Department of Critical Care Medicine, National Cancer Center, Goyang, 10408 South Korea; 2 Department of Innovative Technology, National Cancer Center, Goyang, 10408 South Korea; 3 Division of Convergence Technology, National Cancer Center, Goyang, 10408 South Korea; 4 Healthcare AI Team, National Cancer Center, Goyang, 10408 South Korea; 5 Department of Nursing, National Cancer Center, Goyang, 10408 South Korea; 6 Information Technology Team, National Cancer Center, Goyang, 10408 South Korea; 7 Information Security Team, National Cancer Center, Goyang, 10408 South Korea

**Keywords:** Monitoring system, Rapid response system, Vital sign, Wireless system, Workload

## Abstract

While wireless vital sign monitoring is expected to reduce the vital sign measurement time (thus reducing the nursing workload), its impact on the rapid response system is unclear. This study compared the time from vital sign measurement to recording and rapid response system activation between wireless and conventional vital sign monitoring in the general ward, to investigate the impact of wireless vital sign monitoring system on the rapid response system. The study divided 249 patients (age > 18 years; female: 47, male: 202) admitted to the general ward into non-wireless (n = 101) and wireless (n = 148) groups. Intervals from vital sign measurement to recording and from vital sign measurement to rapid response system activation were recorded. Effects of wireless system implementation for vital sign measurement on the nursing workload were surveyed in 30 nurses. The interval from vital sign measurement to recording was significantly shorter in the wireless group than in the non-wireless group (4.3 ± 2.9 vs. 44.7 ± 14.4 min, *P* < 0.001). The interval from vital sign measurement to rapid response system activation was also significantly lesser in the wireless group than in the non-wireless group (27.5 ± 12.9 vs. 41.8 ± 19.6 min, *P* = 0.029). The nursing workload related to vital sign measurement significantly decreased from 3 ± 0.87 to 2.4 ± 9.7 (*P* = 0.021) with wireless system implementation. Wireless vital sign monitoring significantly reduced the time to rapid response system activation by shortening the time required to measure the vital signs. It also significantly reduced the nursing workload.

## Introduction

The purpose of a rapid response system (RRS) is to assist in the early detection of exacerbations of inpatient conditions and to provide appropriate treatment in order to lower in-hospital cardiopulmonary arrests [1]. An RRS consists of afferent and efferent limbs. The afferent limb is used to detect the exacerbation of patients’ condition while the efferent limb is to provide appropriate treatment in accordance with established criteria for the screened patients [2, 3]. Since the RRS has been reported to be associated with reduced in-hospital cardiopulmonary arrests, many studies have focused on examining and improving the roles of the efferent limb of the RRS [4], and in recent years, there has been a growing interest in RRS quality improvement through the improvement of the afferent limb [5, 6].

As vital signs begin to change before a patient’s condition exacerbates, their accurate and timely measurement is critical. However, the early detection of exacerbation is difficult in the general ward, as high nursing workloads hinder the continuous monitoring of patients [7]. Also, the current intermittent vital sign check system in the general ward is related to detection failure [8, 9] This detection failure causes deterioration of the quality of the afferent limb of RRS [10].

A wireless vital sign monitor automatically records the measured vital signs in the electronic medical records (EMR) without requiring physical input, thereby reducing the time and effort required to measure the vital signs. Several studies have highlighted the usefulness of a wireless vital sign monitoring system [11–13]. In addition, there are increasing reports of clinical deterioration detection in the general ward using continuous and wireless systems, but its contribution to the quality improvement of the afferent limb of the RRS has rarely been examined. [14, 15]

In the present study, we investigated the effects of a wireless vital sign monitoring system on the RRS by examining the intervals from vital sign measurement to recording and from vital sign measurement to RRS activation in the general ward.

## Methods

Patients admitted to a general ward between January and November 2020 were enrolled in this prospective study. Informed consent was obtained from all the participants. Information about the patients’ sex, age, treatment details, time at vital sign measurement and recording, and treatment outcomes was collected. We excluded patients who were admitted for preoperative examination or chemotherapy, who agreed to a do-not-resuscitate order, and who were ineligible for screening for the RRS. This study was approved by the Institutional Review Board of the National Cancer Center (No. NCC2019-0215).

**Fig. 1:**
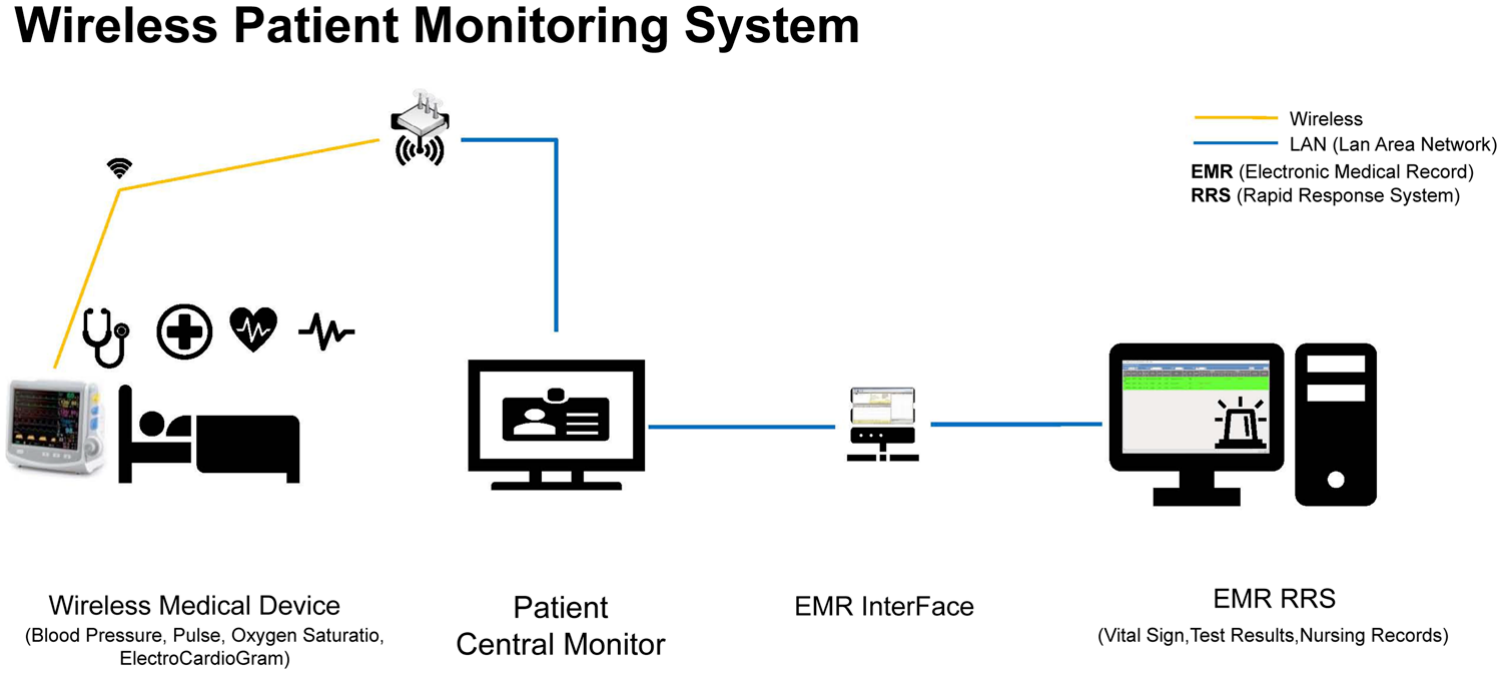
Wireless patient monitoring system: The assigned nurses measure the blood pressure, pulse rate, and body temperature, and the values are directly transmitted to the electronic medical records (EMR)

**Fig. 2:**
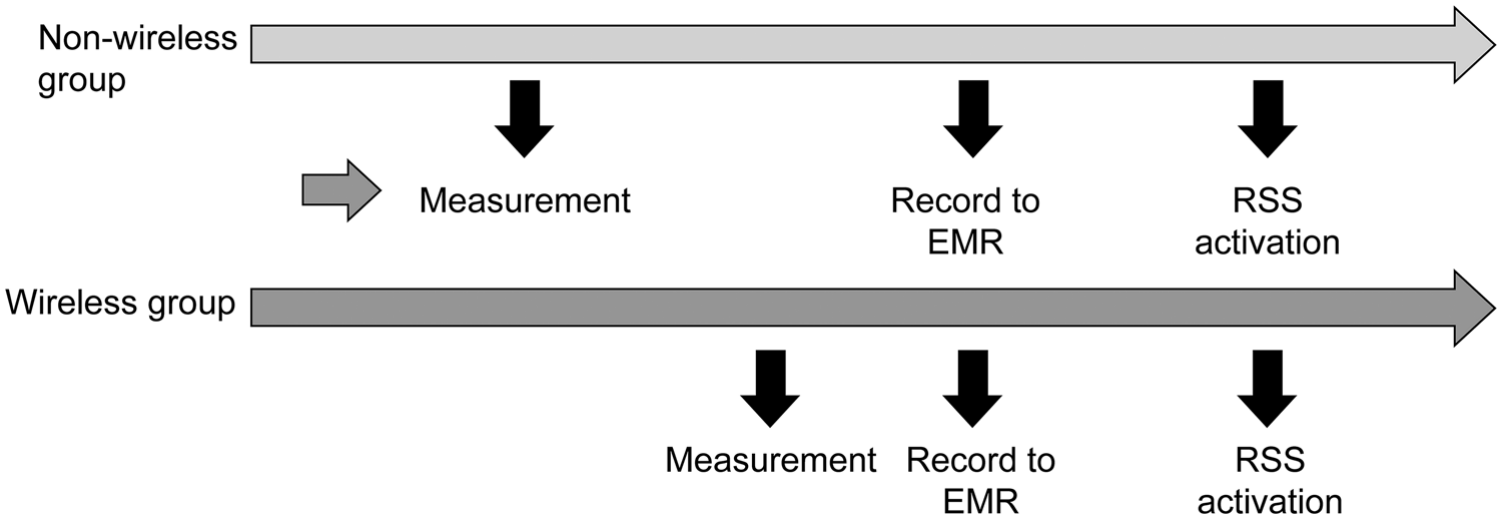
Study outline: (**1**) The time from the start of vital sign monitoring to electronic medical records (EMR) recording is measured. (**2**) For patients for whom the rapid response system (RRS) is activated, the time from the start of vital sign measurement to the examination of the patient by the RRS team is measured

A wireless vital sign monitoring system was implemented in May 2020 at our institution. Patients admitted before the implementation were assigned to the non-wireless group; nurses measured the vital signs of the 5–7 patients assigned to them per duty, and manually recorded them in the EMR. For patients in the wireless group, the assigned nurses measured the blood pressure, pulse rate, and body temperature, and the values were directly transmitted to the EMR (Fig. 1). The respiratory rate (breaths per unit time) was measured by the nurses and manually entered into the EMR. The time from the start of vital sign monitoring to EMR recording was measured, and for patients for whom the RRS was activated, the time from the start of vital sign measurement to examination of the patient by the RRS team was defined as the interval from vital sign measurement to RRS activation (Fig. 2). There were two ways to activate the RRS team. The first involved the direct recording of the vital signs in the EMR and their subsequent registration in the RRS screening, and the second involved the health care providers in the general ward directly contacting the RRS team. If the patient continued to be treated in the general ward, the RRS team conducted several rounds, depending on the patient’s condition, and the number of RRS rounds was recorded for both groups.

To examine the reduction in the nursing workload as a result of the implementation of the wireless monitoring system, a questionnaire was administered to survey the proportion of vital sign measurements in the total nursing workload (10%, 20%, 30%, 40%, 50%, and ≥ 50%) and the workload of vital sign measurement (1 for the lowest workload to 5 for the highest workload). The questionnaire was administered to the same nursing population (n = 30) before and after the implementation of the wireless monitoring system.

Categorical variables were compared using the Pearson χ2 test, and continuous variables were compared using the *t*-test or the Mann–Whitney *U*-test, as appropriate. All values are expressed as mean ± standard error of the mean or as median (range), as appropriate. Statistical significance was set at *P* < 0.05.

## Results

### Patient characteristics

Of the 249 patients included, 101 were in the non-wireless group and 148 were in the wireless group. There were no significant differences in the sex, age, or reason for admission between the two groups. There were also no significant differences in the medical department visits and cancer stages between the two groups.

Regarding treatment outcomes, there were no significant differences in RRS activation, intensive care unit admission, and mortality between the two groups. There was one case of cardiac arrest in the wireless group, but the difference between the two groups was not statistically significant (Table 1).

**Table 1:** Patient characteristics

	**Non-wireless group** **(N = 101)**	**Wireless group** **(N = 148)**	***P*-value**
Sex			0.18
Male	86 (85.1%)	116 (78.4%)	
Female	15 (14.9%)	32 (21.6%)	
Age (years)	62.7 ± 11.5	63.4 ± 10.6	0.14
Reason for admission			0.09
Surgery	37 (36.6%)	38 (25.7%)	
Chemotherapy	27 (26.7%)	36 (24.3%)	
Supportive care	37 (36.6%)	74 (50.0%)	
Department			0.08
Internal medicine	62 (60.2%)	108 (71.5%)	
Surgery	41 (39.8%)	43 (28.5%)	
Cancer treatment			0.26
Curative	42 (40.8%)	72 (48.0%)	
Palliative	61 (59.2%)	78 (52.0%)	
RRS activation	15 (14.9%)	22 (14.9%)	0.1
Adverse event			
ICU admission	3 (3.0%)	5 (3.4%)	0.86
Cardiac arrest	0	1 (0.7%)	> 0.99
Mortality	5 (5.0%)	9 (6.1%)	0.70

Values are presented as mean ± SD or as n (%)

*ICU* intensive care unit, *RRS* rapid response system

### Time interval between vital sign measurement and rapid response system activation

The interval between vital sign measurement and EMR recording was significantly shorter in the wireless group (wireless group vs. non-wireless group: 4.3 ± 2.9 min vs. 44.7 ± 14.4 min, *P* < 0.001), and the interval between vital sign measurement and RRS activation was also significantly shorter in the wireless group (wireless group vs. non-wireless group: 27.5 ± 12.9 min vs. 41.8 ± 19.6 min, *P* = 0.03). The number of RRS team rounds per patient tended to be higher in the wireless group (wireless group vs. non-wireless group: 5.2 ± 2.5 min vs. 4.1 ± 2.7 min, *P* = 0.83 (Table 2).

**Table 2:** Time interval from vital sign measurement to recording and rapid response system activation

	**Non-wireless group** **(N = 101)**	**Wireless group** **(N = 148)**	***P*-value**
Interval from vital sign measurement to recording (min)	44.7 ± 14.4	4.3 ± 2.9	< 0.001
Interval from vital sign measurement to RRS activation (min)	41.8 ± 19.6	27.5 ± 12.9	0.03
Number of RRS rounds per patient	4.1 ± 2.7	5.2 ± 2.5	0.83

Values are presented as mean ± SD

*RRS* rapid response system

### Questionnaire about the workload due to vital sign measurement

**Fig. 3:**
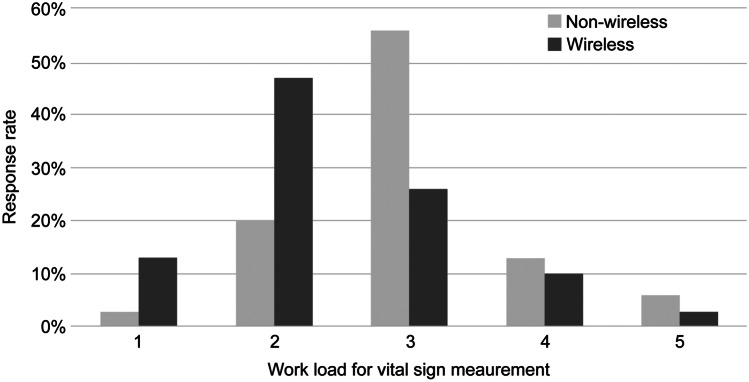
Questionnaire for comparing the workload between the non-wireless and wireless groups

A questionnaire was administered to the same nurse group before and after the implementation of the wireless vital sign monitoring system. The proportion of vital sign measurement in the total nursing workload did not significantly differ before (30.6 ± 10.4%) and after (28.0 ± 8.4%) the implementation of the wireless monitoring system (*P* = 0.28). However, the workload due to vital sign measurement significantly decreased from 3 ± 0.87 to 2.4 ± 9.7 (*P* = 0.02) (Fig. 3).

## Discussion

In the present study, the application of the wireless vital sign monitoring system not only shortens the time needed to measure the vital signs, but also improves the quality of the RRS by preventing the delay in RRS activation.

In response to reports that a delay in the delivery of appropriate treatment when a patient’s condition is exacerbated, is associated with prognosis, and there is a growing interest regarding the reduction of time from exacerbation of the patient’s condition to the delivery of appropriate treatment through the RRS [16–18]. While some studies have examined the usefulness of wireless vital sign monitoring [8–10], the present study differs from those studies because it confirmed that a wireless system not only shortens the time needed to measure vital signs, but also improves the quality of the RRS by preventing the delay in RRS activation.

Unlike in the intensive care unit (ICU), vital signs are only intermittently monitored in the general ward, with long intervals between monitoring. Failure to detect any exacerbation in a timely manner, with consequent delay in the activation of the afferent limb, has been associated with increased in-hospital cardiac arrests and mortality [5, 19]. In particular, cardiopulmonary arrest in cancer patients is frequently accompanied by sepsis and has poor outcomes [20]. Reducing the time taken to measure vital signs is crucial for a prompt diagnosis of sepsis and delivery of appropriate treatment, including broad-spectrum antibiotics [21, 22]. This suggests that the shortening of vital sign measurement and reduction of the relevant nursing workload via the implementation of a wireless system will be associated with improved outcomes with RRS.

In the present study, the nursing workload related to vital sign measurement after the implementation of a wireless system was examined via a survey. Increased nursing workload has significantly been associated with patient harm, including medication error and bed-related falls [23, 24]. Furthermore, increased nursing workload has been reported to predict healthcare-associated infections, including central line-associated blood stream infection and ventilator-associated pneumonia [25]. Considering that increasing the nursing staffing and reducing the work hours are difficult, lowering the nursing workload by reducing the time taken to measure vital signs via the implementation of a wireless system could be a solution to increasing nursing work efficiency. The proportion of vital sign tasks of the total nursing work was higher than expected. Compared to the ICU or other special units, the general ward has fewer nursing tasks other than the measurement, which they may have considered to be performed more than other tasks. Moreover, the fact that vital sign measurement make up a higher proportion of nursing work than expected suggests that further research is needed to improve the vital sign measurement and EMR recording process in a more efficient way.

Currently, wireless-based medical devices are considered very vulnerable with regards to authentication, transmission, storage data protection, personal information protection, platform protection, and device access control [26]. Overall, it is understood that the existing technology that was designed to prioritize system configuration has limitations with regards to the protection of the safety and privacy of patients, in addition to security issues related to the Internet of things (IoT) medical devices. To overcome these limitations, security and privacy protection systems should be considered from the design/developmental stage of the IoT medical devices and services, and infringement factors that may occur during IoT medical device development should be investigated in advance by benchmarking the privacy impact assessment [27]. Despite the related problems of such a wireless-based medical device or IoT medical device, there are recent evidence that it is being used as a standard of care system in several hospitals [14, 15].

In the present study, there were some important limitations. First, this study was designed as a before-and-after study, which has its limitations [28, 29]; however, the effect of the vital sign measurement process on the study results might be limited. There was a difference between the number of male and female patients in this study. However, the difference had little effect on the limited vital sign measurement time. Second, the implemented wireless system measured the blood pressure, pulse rate, and body temperature, while nurses manually entered their measurements of respiratory rate. Respiratory rates are reported to be highly vulnerable to error or variations depending on the observer [30], thereby highlighting the need for diagnostic tools that can accurately measure respiratory rates. Another limitation of this study was that it was difficult to reflect the differences between a wireless system and a non-wireless system when the RRS was activated through direct contact by healthcare providers.

It is to be expected that a wireless vital sign monitoring system could be feasible as a standard patient care system in many hospitals by using that to replace the existing vital sign measurement method, as indicated above. Further studies are needed on the effect of the wireless vital sign monitoring system applied to RRS on the reduction of in-hospital cardiac arrests and mortality.

## Conclusions

A wireless vital sign monitoring system can significantly shorten the time to RRS activation by reducing the time needed to measure the vital signs. Furthermore, the system also contributes to reducing the nursing workload.

## Data Availability

Not applicable.

## References

[1] Jones DA, DeVita MA, Bellomo R (2011). Rapid-response teams. N Engl J Med.

[2] Chen J, Ou L, Hillman K, Flabouris A, Bellomo R, Hollis SJ, Assareh H (2014). The impact of implementing a rapid response system: a comparison of cardiopulmonary arrests and mortality among four teaching hospitals in Australia. Resuscitation.

[3] Chen J, Ou L, Hillman KM, Flabouris A, Bellomo R, Hollis SJ, Assareh H (2014). Cardiopulmonary arrest and mortality trends, and their association with rapid response system expansion. Med J Aust.

[4] Oh TK, Kim S, Lee DS, Min H, Choi YY, Lee EY, Yun MA, Lee YJ, Hon PS, Kim K, Do SH, Hwang JW, Song IA (2018). A rapid response system reduces the incidence of in-hospital postoperative cardiopulmonary arrest: a retrospective study. Can J Anaesth.

[5] Barwise A, Thongprayoon C, Gajic O, Jensen J, Herasevich V, Pickering BW (2016). Delayed rapid response team activation is associated with increased hospital mortality, morbidity, and length of stay in a tertiary care institution. Crit Care Med.

[6] Tirkkonen J, Skrifvars MB, Tamminen T, Parr MJA, Hillman K, Efendijev I, Aneman A (2020). Afferent limb failure revisited – A retrospective, international, multicentre, cohort study of delayed rapid response team calls. Resuscitation.

[7] Thomas EJ, Studdert DM, Burstin HR, Orav EJ, Zeena T, Williams EJ, Howard KM, Weiler PC, Brennan TA (2000). Incidence and types of adverse events and negligent care in Utah and Colorado. Med Care.

[8] Saab R, Wu BP, Rivas E, Chiu A, Lozovoskiy S, Ma C, Yang D, Turan A, Sessler DI (2021). Failure to detect ward hypoxaemia and hypotension: contributions of insufficient assessment frequency and patient arousal during nursing assessments. Br J Anaesth.

[9] Taenzer AH, Spence BC (2018). The afferent limb of rapid response systems. Crit Care Clin.

[10] Eddahchouri Y, Koeneman M, Plokker M, Brouwer E, van de Belt TH, van Goor H, Bredie SJ (2021). Low compliance to a vital sign safety protocol on general hospital wards: A retrospective cohort study. Int J Nurs Stud.

[11] Posthuma LM, Downey C, Visscher MJ, Ghazali DA, Joshi M, Ashrafian H, Khan S, Darzi A, Goldstone J, Preckel B (2020). Remote wireless vital signs monitoring on the ward for early detection of deteriorating patients: a case series. Int J Nurs Stud.

[12] Boatin AA, Wylie BJ, Goldfarb I, Azevedo R, Pittel E, Ng C, Haberer JE (2016). Wireless vital sign monitoring in pregnant women: a functionality and acceptability study. Telemed J E Health.

[13] Weenk M, Koeneman M, van de Belt TH, Engelen LJLPG, van Goor H, Bredie SJH (2019). Wireless and continuous monitoring of vital signs in patients at the general ward. Resuscitation.

[14] Eddahchouri Y, Peelen R, van Koeneman M, Touw HRW, van Goor H, Bredie SJH (2022). Effect of continuous wireless vital sign monitoring on unplanned ICU admissions and rapid response team calls: a before-and-after study. Br J Anaesth.

[15] Heller AR, Mees ST, Lauterwald B, Reeps C, Koch T, Weitz J (2020). Detection of deteriorating patients on surgical wards outside the ICU by an Automated MEWS-based early warning system with paging functionality. Ann Surg.

[16] Buist MD, Jarmolowski E, Burton PR, Bernard SA, Waxman BP, Anderson J (1999). Recognising clinical instability in hospital patients before cardiac arrest or unplanned admission to intensive care: a pilot study in a tertiary-care hospital. Med J Aust.

[17] Reardon PM, Fernando SM, Murphy K, Rosenberg E, Kyeremanteng K (2018). Factors associated with delayed rapid response team activation. J Crit Care.

[18] Cho KJ, Kwon O, Kwon JM, Lee Y, Park H, Jeon KH, Kim KH, Park J, Oh BH (2020). Detecting patient deterioration using artificial intelligence in a rapid response system. Crit Care Med.

[19] Park J, Lee YJ, Hong SB, Jeon K, Moon JY, Kim JS, Kang BJ, Ahn JJ, Lee DH, Park J, Cho JH, Lee SM (2021). The association between hospital length of stay before rapid response system activation and clinical outcomes: a retrospective multicenter cohort study. Respir Res.

[20] Abou Dagher G, El Khuri C, Chehadeh AA, Chami A, Bachir R, Zebian D, Bou Chebl R (2017). Are patients with cancer with sepsis and bacteraemia at a higher risk of mortality? A retrospective chart review of patients presenting to a tertiary care centre in Lebanon. BMJ Open.

[21] Seymour CW, Gesten F, Prescott HC, Friedrich ME, Iwashyna TJ, Phillips GS, Lemeshow S, Osborn T, Terry KM, Levy MM (2017). Time to treatment and mortality during mandated emergency care for sepsis. N Engl J Med.

[22] Levy MM, Evans LE, Rhodes A (2018). The surviving sepsis campaign bundle. 2018 update. Intensive Care Med.

[23] Garrett C (2008). The effect of nurse staffing patterns on medical errors and nurse burnout. AORN J.

[24] Stone PW, Mooney-Kane C, Larson EL, Horan T, Glance LG, Zwanziger J, Dick AW (2007). Nurse working conditions and patient safety outcomes. Med Care.

[25] Shekelle PG (2013). Nurse-patient ratios as a patient safety strategy: a systematic review. Ann Intern Med.

[26] Hatzivasilis G, Soultatos O, Ioannidis S, Verikoukis C, Demetriou G, Tsatsoulis C. (2019) Review of security and privacy for the internet of medical things (IoMT), 2019. 15th International Conference on Distributed Computing in Sensor Systems (DCOSS), Santorini Island, Greece, pp 457–464. 10.1109/DCOSS.2019.00091

[27] Clarke R (2009). (2009) Privacy impact assessment: its origins and development. Comput Law Secur Rev.

[28] Nedel WL, da Silveira F (2016). Different research designs and their characteristics in intensive care. Rev Bras Ter Intensiva.

[29] Choi SW, Wong GTC (2018). Quality improvement studies - pitfalls of the before and after study design. Anaesthesia.

[30] Philip KE, Pack E, Cambiano V, Rollmann H, Weil S, O'Beirne J (2015). The accuracy of respiratory rate assessment by doctors in a London teaching hospital: a cross-sectional study. J Clin Monit Comput.

